# Primary Sjogren’s Syndrome Presenting As Pleuritis and Pleural Effusion

**DOI:** 10.7759/cureus.75019

**Published:** 2024-12-03

**Authors:** Ashwin Varkey, Kevin Shayani, Viera Lakticova

**Affiliations:** 1 Medicine, Lenox Hill Hospital, New York, USA; 2 Pulmonary and Critical Care Medicine, Lenox Hill Hospital, New York, USA

**Keywords:** autoimmune disorder, pleurisy, rare cause of pleural effusion, sjogrens syndrome, video-assisted thoracoscopic surgery (vats)

## Abstract

Sjogren’s syndrome is an autoimmune condition characterized by infiltration of exocrine glands but, in rare cases, can have extraglandular involvement with pleural effusion being an exceedingly rare form. Here we present a case of Sjogren’s pleuritis resulting in pleural effusion, a rare initial presentation for Sjogren’s syndrome. A woman in her 20s presented to the emergency department after a recent hospitalization for pneumonia, pleural effusion, and pulmonary embolism. She presented to the hospital after discharge with pleuritic chest pain, dyspnea, and a productive cough. She was subsequently found to have a large left pleural effusion that necessitated left video-assisted thoracoscopic surgery with pleural biopsy and decortication of the lung. Infectious etiologies were ruled out during hospitalization. The patient’s pleural fluid studies revealed lymphocytic predominance, and biopsy results were consistent with the autoimmune origin, more specifically, Sjogren’s serositis. While Sjogren’s syndrome is an autoimmune disorder most commonly affecting lacrimal and salivary gland function, it is important to consider extra-glandular involvement in patients with positive serologies and unexplained phenomena. This manuscript aims to provide information regarding diagnostic tools and criteria to arrive at a diagnosis and effectively treat pleural effusion secondary to Sjogren's serositis.

## Introduction

Sjogren's disease is an autoimmune condition with primarily exocrine glandular manifestations resulting in dry mouth and mucous membranes, but extra-glandular manifestations exist, including involvement of the kidneys, GI tract, and lungs [1]. While pulmonary parenchymal involvement of Sjogren’s syndrome is a known association, pleural involvement is rare, with most of the literature existing in the form of case reports and series. In patients with unexplained pleural effusion and positive serologies for Sjogren’s syndrome, pleural involvement of Sjogren’s must be considered. Sampling of the pleural fluid will reveal lymphocytic predominance, with gram stain, cultures, and the remainder of the studies largely negative for an alternative etiology [2]. Pleural biopsy is required to simultaneously exclude other causes such as granulomatous inflammation and disease [3]. Pleural biopsy will also reveal chronic inflammation and lymphocytic infiltration in cases associated with Sjogren’s [4]. This report aims to highlight the challenges in diagnosing this condition, including all relevant workup and diagnostic findings. Given the rarity of the condition as well as the paucity of literature on the matter, prompt diagnosis is important, as treatment of the underlying autoimmune condition is often required to prevent recurrence [1].

## Case presentation

A female in her mid-20s initially presented to the emergency department two weeks after prior hospitalization due to worsening dyspnea, productive cough, and pleuritic chest pain for one day. The patient had a past medical history significant for antiphospholipid syndrome, polycystic ovarian syndrome, and obesity. She had a recent hospitalization and was found to have a pulmonary embolism, pneumonia, and a moderate left-sided pleural effusion. She was hospitalized for three days during a previous visit. The patient was discharged on oral amoxicillin-clavulanic acid for pneumonia, which was diagnosed via imaging and clinical symptoms, and warfarin for her pulmonary embolism. The patient finished her prescribed antibiotic outpatient and was compliant with warfarin for her pulmonary embolism. The patient’s family history was significant for psoriasis in her father and diabetes mellitus in her mother. The patient denied ever smoking cigarettes or using recreational drugs. The patient reported social alcohol use, about one to three alcoholic mixed drinks per weekend. Upon interview, she did not report any fevers, chills, changes in bowel habits, new arthralgias, or new rash. Our patient did report dryness in her eyes. The patient denied any sick contacts. Upon initial physical exam, the patient was afebrile, with a heart rate of 88 beats per minute, blood pressure of 141/76 mmHg, and was saturating 94% on room air. The patient was alert, oriented, and in no acute distress upon presentation. The head, eyes, ear, nose, and throat exam was insignificant. Pulmonary examination was only notable for diminished breath sounds on the left side. Cardiac examination was insignificant. The patient’s extremities were warm and well-perfused, with no edema. Of note, the patient reported persistent brown sputum production since the previous discharge, which was 15 days prior, despite the completion of an oral amoxicillin-clavulanic acid course. Laboratory findings were significant for a white blood cell count of 12,130 per microliter (4,000-11,000 per microliter), hemoglobin of 12.1 grams per deciliter (12.0-16.0 g/dL), and platelets of 308,000 per microliter (150,000-400,000). Her international normalized ratio was 2.34 (0.8-1.1), prothrombin time was 2.81 seconds (11.5-13.5 seconds), and activated partial thromboplastin time was 103.4 seconds (21-35 seconds). The remainder of blood chemistries, liver function tests, and urinalysis were within normal limits. The patient had an elevated CRP of 143.8 mg/L (0-4 mg/L) and erythrocyte sedimentation rate (ESR) of 74 mm/hr (<15 mm/hr). Due to concern for underlying autoimmune etiology given past medical history of antiphospholipid syndrome, further tests were ordered. The patient’s ANA was positive at 1:320 in a speckled pattern (<1:80). Double-stranded antibody, Smith antibody, and anti-ribonuclear protein were all negative. Anti-SSB was elevated at 5.9 (<0.9), and anti-SSA was elevated and greater than 8.0 (<0.9). C-ANCA, p-ANCA, and atypical ANCA antibodies were negative.

**Figure 1:**
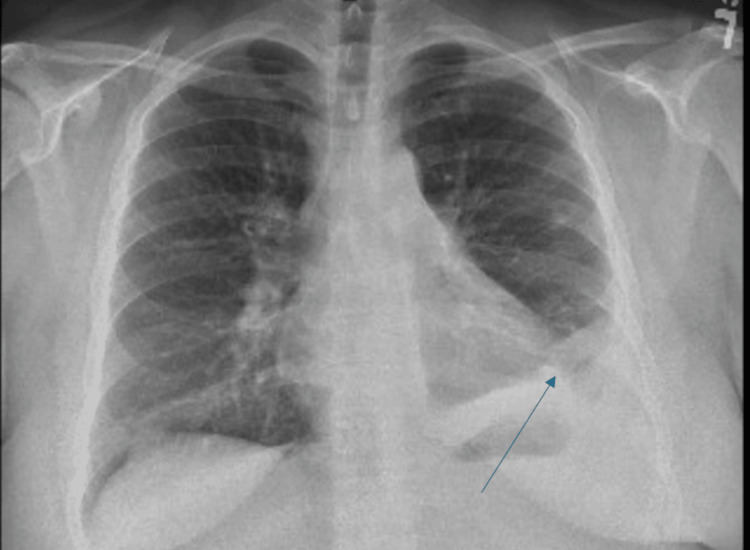
Arrow pointing to area on chest X-ray of opacification

A chest X-ray was done on admission, which revealed left hemithorax opacification (Figure 1).

**Figure 2:**
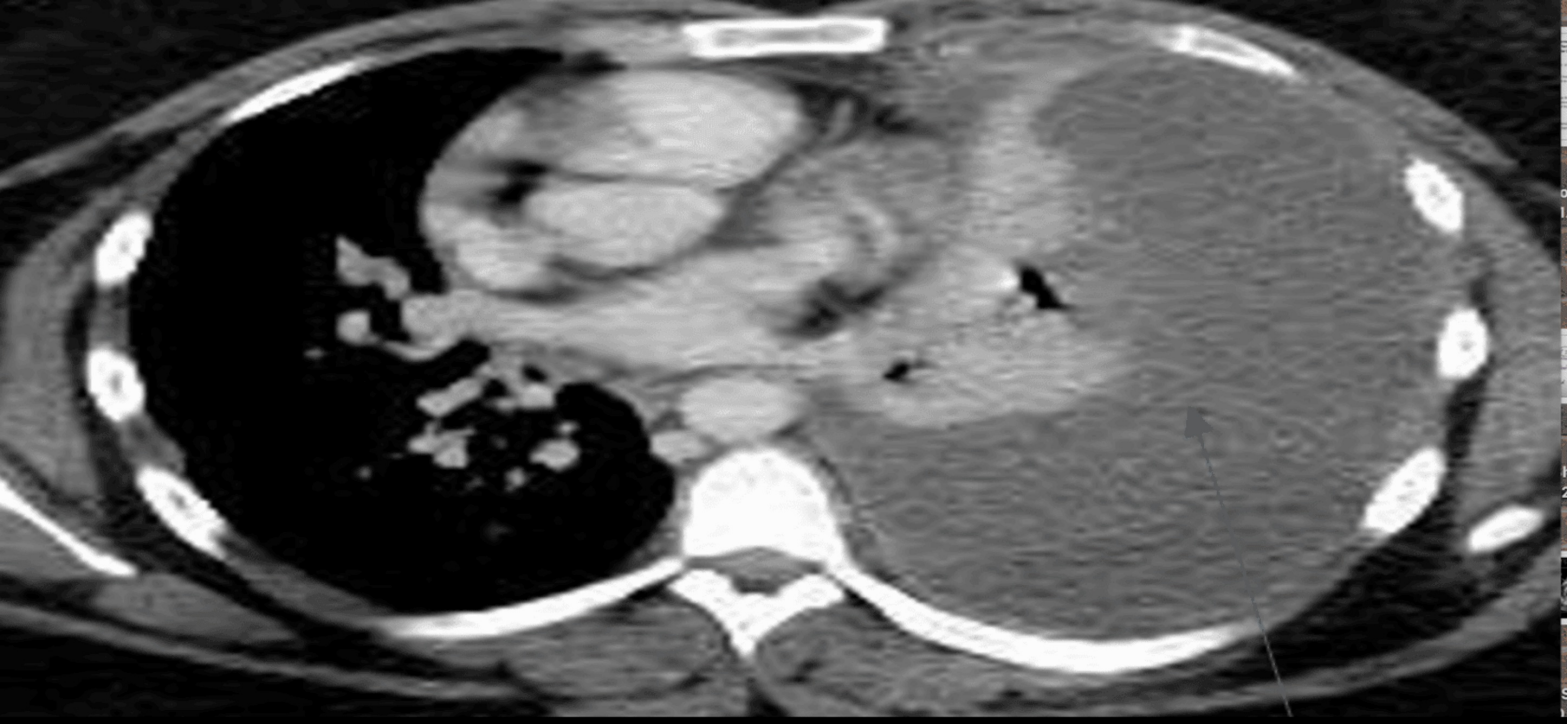
Arrow pointing to large L-sided pleural effusion upon admission to the hospital

A computed tomography (CT) scan of the chest with intravenous contrast was done, which revealed left lung compressive atelectasis due to large pleural effusion (Figure 2).

**Figure 3:**
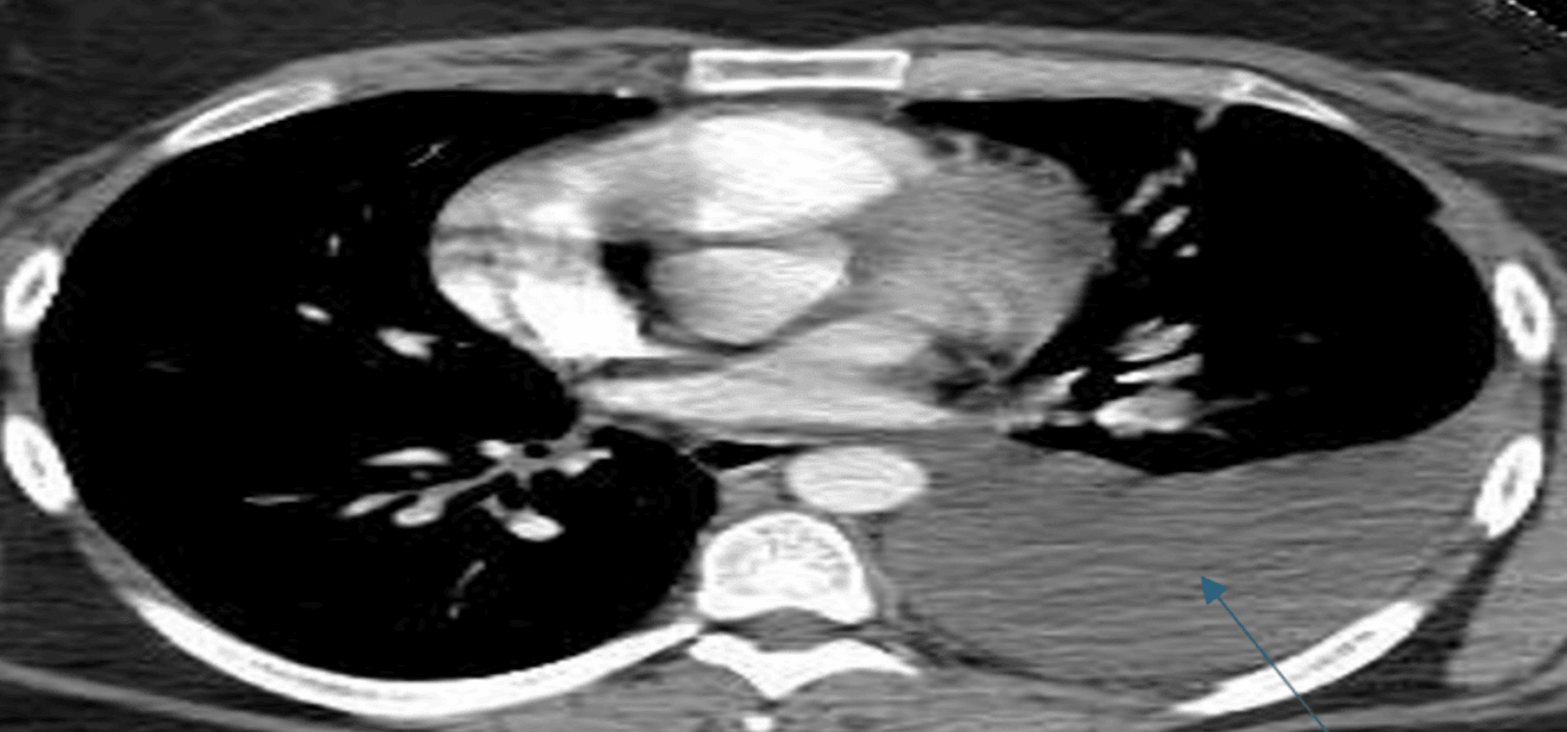
Arrow pointing to previous CT scan done three weeks ago with left pleural effusion

This pleural effusion increased in size from moderate to large from the previous CT scan of the chest, which was done during prior admission three weeks ago (Figure 3).

**Figure 4:**
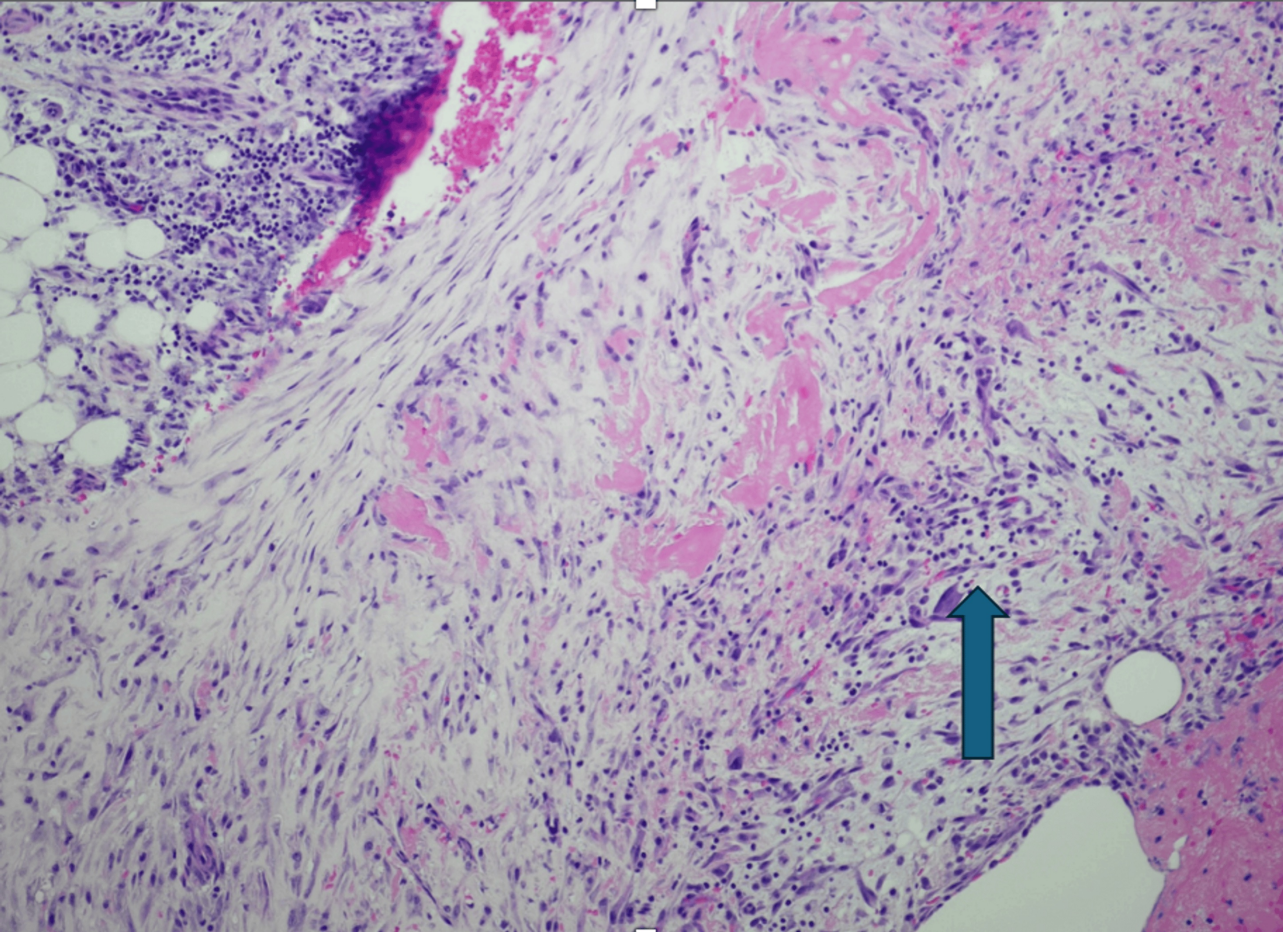
Arrow pointing to inflamed pleura with predominant lymphoplasmocytic infiltrate

She underwent pigtail catheter placement, with pleural fluid analysis revealing high cellularity, with total cells of 1979 (<1000 cells/uL), red blood cell count of 117,000 (<10,000/uL), 22% neutrophils (no reference range), 74% lymphocytes (no reference range), and 4% monocytes/macrophages (no reference range). The pH of the pleural fluid was 7.38, and adenosine deaminase was within normal limits of 19 (0-30 U/L). The normal adenosine deaminase effectively ruled out tuberculosis as the cause of lymphocyte-rich pleural effusion. The pleural fluid culture did not grow any organisms, and cytology was negative for malignant cells. Acid-fast bacilli were not isolated from the pleural fluid after six weeks. She underwent left video-assisted thoracoscopic surgery, pleural biopsy, and decortication of the lung, with pleural biopsies revealing chronic inflammatory changes and lymphocytic infiltration (Figure 4).

There was no evidence of granulomatous disease. A diagnosis of Sjogren’s pleuritis was made, and she was discharged with continued multidisciplinary follow-up. Our patient has had no further exacerbations of Sjogren's pleuritis to this date and has continued to follow up with rheumatology for her Sjogren's syndrome.

## Discussion

Sjogren’s syndrome is an autoimmune disorder affecting the exocrine glands and is histologically associated with lymphocytic infiltration [1]. It is characterized by diminished lacrimal and salivary gland function, and common symptoms include dryness of the mouth and eyes [5]. Up to one-half of Sjogren's individuals are noted to have extra-glandular involvement in organs such as the kidneys, lungs, GI tract, and nervous system [6]. About 10% to 20% of patients can have pulmonary involvement, with the most common manifestation being a dry cough secondary to glandular involvement of the airways [7]. While pulmonary involvement, particularly in the form of interstitial lung disease, is a known association with Sjogren’s disease, pleural involvement is exceedingly rare, with most literature existing in the form of case reports and series [7]. As of 2020, less than 1% of primary Sjogren’s syndrome cases have been associated with pleural involvement [2]. In cases of pleural involvement, the fluid is often rich in lymphocytes [8].

The pathophysiology of Sjogren’s syndrome is based on lymphocyte-rich mononuclear cells infiltrating exocrine glandular tissue [1]. Histology is predominantly T lymphocytes, and immune complex deposition in skin, joints, and organs can result in systemic vasculitis [5]. Typical biopsy findings reveal aggregates of usually greater than 50 lymphocytes in conjunction with some macrophages and plasma cells [8]. Diagnosis of Sjogren’s does not revolve around a single test and instead relies on a combination of clinical and laboratory findings after the exclusion of alternative causes [9]. The diagnosis can be made if there are objective findings of ocular and/or oral dryness with the presence of underlying autoimmunity (with a positive anti-SSA in the presence or absence of anti-SSB) and a positive biopsy showing lymphocytic infiltration [1]. Our patient, upon discussion, reported ocular dryness and had positive anti-SSA as well as a positive biopsy with lymphocytic infiltration. Secondary Sjogren’s can also be seen in patients with a confirmed underlying autoimmune condition such as lupus or mixed connective tissue disease where there is lacrimal and salivary gland dysfunction [2].

In our case, we illustrate a female in her 20s with recurrent pleural effusion of initially unknown etiology. Left video-assisted thoracoscopic surgery with pleural biopsy and decortication of the lung was required with short-term resolution of her pleural effusion. Biopsy and serological markers confirmed Sjogren’s syndrome as the most likely etiology of pleural effusion. Sjogren’s pleural effusions will typically be lymphocyte predominant, which distinguishes them from other autoimmune effusions such as those of lupus, which will display a neutrophil-rich exudate [2]. In a patient with recurrent or worsening pleural effusion and known underlying autoimmunity, it is reasonable to consider the underlying autoimmune condition as responsible [4]. In addition to serologies to work up rheumatic conditions, chest imaging (particularly CT) and diagnostic pleural fluid studies can be of additional yield - particularly in ruling out infectious or granulomatous etiologies [3]. Pleural involvement in primary Sjogren’s should raise the concern of an overlap syndrome, as many of these patients may have underlying rheumatoid arthritis or systemic lupus that is not yet diagnosed [3]. In these cases, the Sjogrens would then be classified as secondary, and patients should seek treatment for their underlying primary rheumatologic disorder [5]. Most cases of Sjogren’s associated pleuritis resolve spontaneously, with some case reports highlighting the role of corticosteroids [1]. Similarly, only case reports exist in the literature regarding surgical management and diagnosis with video-assisted thoracoscopic surgery and decortication [8]. Our case illustrated a rare case of Sjogren's pleuritis and illustrates the importance of a broad workup and differential diagnosis in this unique situation.

## Conclusions

This case highlights the important association between primary Sjogren’s syndrome and pleural involvement, specifically presenting as recurrent pleural effusion. While pleural effusion in Sjogren’s is uncommon, it is important to consider this possibility in patients, especially when pleural fluid analysis reveals a lymphocyte-predominant exudate. Our patient’s presentation illustrates the importance of a comprehensive diagnostic approach, including serological testing and imaging, to identify Sjogren’s as a potential etiology of pleural effusion. Furthermore, while most cases of Sjogren’s-associated pleuritis resolve spontaneously or with corticosteroids, this case also illustrates the rare necessity of surgical intervention, such as video-assisted thoracoscopic surgery and pleural decortication, for diagnosis and management. Given the potential for overlap with other autoimmune diseases, clinicians should consider secondary autoimmune conditions in patients with primary Sjogren’s syndrome, which may influence management. In conclusion, this case contributes to the limited literature on pleural involvement in Sjogren’s syndrome and reinforces the need for a thorough differential diagnosis in patients presenting with recurrent pleural effusions.
